# Associations between Total Atherosclerosis Burden of Baroreceptor-Resident Arteries and ECG Abnormalities after Acute Ischemic Stroke

**DOI:** 10.3390/brainsci14050505

**Published:** 2024-05-16

**Authors:** Zhiyong Fu, Xin Ma, Xiaoxi Zhao, Xiangying Du, Yungao Wan

**Affiliations:** 1 Department of Neurology, Xuanwu Hospital, Capital Medical University, No. 45 Changchun St., Beijing 100053, China; 2 National Clinical Research Center for Geriatric Disorders, Beijing 100053, China; 3 Clinical Center for Cardio-Cerebrovascular Disease, Capital Medical University, Beijing 100053, China; 4 Department of Radiology, Xuanwu Hospital, Capital Medical University, Beijing 100053, China; 5 Department of Cardiology, Xuanwu Hospital, Capital Medical University, Beijing 100053, China

**Keywords:** acute ischemic stroke, atherosclerosis burden, baroreceptor-resident arteries, electrocardiography, heart rate variability

## Abstract

Electrocardiogram (ECG) abnormalities are the most common cardiac complications after acute ischemic stroke (AIS) and predict poor outcomes. The arterial baroreflex is an essential determinant of cardiovascular autonomic regulation, with receptors mainly residing in carotid sinuses and aortic arch. The atherosclerosis of these baroreceptor-resident arteries (BRA) is very common in AIS patients and might impair baroreflex function. However, the associations between the atherosclerosis of BRA and ECG abnormalities after AIS are still unknown. In total, 228 AIS patients within 7 days after onset without a pre-existing heart disease were prospectively recruited. With computed tomography angiography, atherosclerosis conditions in 10 segments of the carotid sinuses and aortic arch were scored and summed as the Total Atherosclerosis Burden of BRA (TAB-BRA), and asymptomatic coronary artery stenosis (ACAS) ≥50% was simultaneously assessed. We performed 12-lead ECG to dynamically detect abnormal repolarization, and 24 h Holter ECG to monitor arrhythmias and heart rate variability (HRV) parameters, which are reliable indicators to assess cardiac autonomic function. We found that TAB-BRA was positively associated with abnormal repolarization (OR 1.09; CI% 1.03–1.16; *p* = 0.003) and serious cardiac arrhythmias (OR 1.08; CI% 1.01–1.15; *p* = 0.021). In addition, TAB-BRA was an important predictor of abnormal repolarization, persisting over 3 days (OR 1.17; CI% 1.05–1.30; *p* = 0.003). However, ACAS ≥ 50% did not relate to these ECG abnormalities. TAB-BRA was negatively correlated with parasympathetic-related HRV parameters. Our results indicated that AIS patients with a high TAB-BRA are more likely to have ECG abnormalities and delayed normalization, which may relate to the decreased cardiac parasympathetic activity, but not the accompanied ACAS ≥ 50%.

## 1. Introduction

Acute ischemic stroke (AIS) can induce a continuum of cardiac complications even without previous cardiac disease. These complications include ECG abnormalities, myocardial injury, and cardiac dysfunction [1]. Particularly, ECG abnormalities, mainly presenting with abnormal repolarization and arrhythmias, are most often observed in up to 70–90% of AIS patients during the first few days after onset, even under antiplatelet, anticoagulation, or interventional treatments, and predict poor outcomes [1,2,3]. The pathophysiological mechanism is complex and seems to be caused by brain–heart axis dysfunction triggered by lesions of the brain’s central autonomic network, leading to a shift in autonomic balance to sympathetic hyperactivity [4]. A previous study found that the autonomic imbalance might persist for 6 months after AIS [5]. Further exploration of factors influencing ECG abnormalities and their differences in duration after AIS could help to gain a deeper understanding of their mechanisms and potential targets for intervention.

The arterial baroreflex, an essential determinant of cardiovascular regulation, delicately regulates cardiac sympathetic and parasympathetic activity, and decreased baroreflex sensitivity can lead to an impaired regulation of blood pressure and heart rate, even arrhythmias [6,7]. Baroreceptors are located in the carotid sinuses and the aortic arch region, which are the most common sites for atherosclerotic lesions [6,8]. Atherosclerosis in these baroreceptor-resident arteries (BRA) may blunt baroreflex sensitivity by reducing wall distensibility, then lead to corresponding changes in parasympathetic and sympathetic nervous activity [9,10]. Notably, nearly 80% of AIS patients had atherosclerosis in BRA [10], but the associations between the atherosclerosis of BRA and ECG abnormalities after AIS have never been studied before.

Finding a method comprehensively evaluating the atherosclerosis of BRA is crucial to elucidate this issue. We had innovatively established an atherosclerosis score to reflect the overall atherosclerosis severity and extent in both carotid sinuses and the aortic arch, namely, the Total Atherosclerosis Burden of BRA (TAB-BRA) [10]. In addition, the heart rate variability (HRV) obtained by a 24 h Holter is often used to indicate the sympathetic and parasympathetic activities and their balance. It is helpful to understand the influence of TBA-BRA on cardiac autonomic function. Concomitantly, the atherosclerosis of brain- and heart-supplying arteries share common risk factors and pathological mechanisms. It is unclear whether the ECG abnormalities after AIS are caused by an accompanied subclinical coronary artery disease—asymptomatic coronary artery stenosis (ACAS) ≥ 50%. Thus, we performed a one-step combined computed tomography angiography (CTA) to assess the atherosclerosis of carotid sinuses, aortic arch, and coronary arteries, facilitating the simultaneous investigation of the associations between TAB-BRA, ACAS ≥ 50%, and ECG abnormalities in AIS patients without a pre-existing heart disease.

## 2. Methods

### 2.1. Study Design and Participants

This was a single-center cross-sectional study. All the procedures complied with the Declaration of Helsinki. This study was approved by the local ethics committee, and written informed consent was provided by all participants. From a prospective registry of patients with ischemic cerebrovascular disease, patients admitted to the stroke unit in Xuanwu Hospital of Capital Medical University from 1 March 2023 to 30 October 2023 were consecutively recruited. Patients were eligible if they were diagnosed with AIS with the confirmation of magnetic resonance imaging (MRI); 18 to 85 years of age; within 7 days after symptom onset; and consented to participate. Individuals were excluded if they had a pre-existing heart disease; or had recently used drugs affecting cardiac electrical instability; or had serious infectious diseases, electrolyte disturbances, and thyrotoxicosis; or had diseases related to autonomic nervous system disorders (e.g., multiple system atrophy and Parkinson’s disease, etc.); or whose data from an ECG and 24 h Holter were incomplete or not interpretable; or had carotid sinuses and aortic arch aneurysm/surgery history; or were unable to undergo CTA, including contrast agent allergy and renal insufficiency. The data supporting the findings of this study are available from the corresponding author for checking the reproducibility of the study results. This study followed the Strengthening the Reporting of Observational Studies in Epidemiology statement [11].

### 2.2. General Characteristics and Stroke Features

Age, sex, body mass index (BMI), smoking status, hypertension, diabetes mellitus, and prior strokewere collected at the time of enrollment through face-to-face interviews, as previously published [12]. The stroke features were evaluated in three aspects: (1) Stroke severity was assessed at admission by neurologists using the National Institutes of Health Stroke Scale (NIHSS). (2) Infarct volumes were manually calculated from the diffusion-weighted imaging (DWI) sequence of brain MRI, using the following formula: a × b × c × d × 0.5, in which a and b mean the largest length and width of the hyperintensity zone, c means the number of sections where the lesion is present, and d means the slice thickness of the sections [13]. (3) Insular involvement was documented by tracing on DWI sequences. To minimize bias, all investigators involved in the stroke feature evaluation were blinded to the other characteristics of the patients.

### 2.3. Assessment of ECG Abnormalities and Heart Rate Variability

A baseline resting standard 12-lead ECG was performed at the time of hospital admission to detect abnormal repolarization, then a repeated ECG was conducted daily for at least 7 days, and whether abnormal repolarization persisted over 3 days was recorded. If an individual’s onset time exceeded 3 days at admission, the initial ECG information within 3 days would be traced back from their first visited medical facilities (such as an ambulance or community outpatient clinic). We also routinely performed a 24 h Holter ECG within 7 days after onset to monitor arrhythmias and assess HRV.

Abnormal repolarization included prolongation in the duration of the corrected QT interval (QTc) and changes in the ST segment and T wave or Q wave, all of which reflect abnormalities in ventricular repolarization [1]. The QT interval was corrected for heart rate using the Bazett formula. A prolonged QTc was defined as ≥450 ms for men and ≥460 ms for women [14]. The definition of abnormalities in the ST segment and T wave or Q wave followed the Recommendations for the Standardization and Interpretation of the Electrocardiogram [14]. All ECGs have been read by cardiologists.

Serious cardiac arrhythmias (SCA) were automatically identified using DM Software (CardioScan, Version 4, Tustin, CA, USA), and manually over-read by cardiologists. SCA included all arrhythmic episodes causing symptoms or meeting one of the following ECG criteria in line with the prospective Stroke–Arrhythmia-Monitoring-Database (SAMBA) study [15]: ventricular flutter/fibrillation, ventricular tachycardia, or new-onset ventricular ectopy; supraventricular tachycardia >130 beats/min; and sinus arrest for >3 s or drops in heart rate below 30 beats/min because of sinoatrial block, second- and third-degree atrioventricular block, or atrial fibrillation.

HRV parameters were used to assess cardiac autonomic function [14]. Two time-domain measures of HRV, including the standard deviation of all normal-to-normal RR intervals (SDNN) and the root mean square of differences in adjacent normal-to-normal RR intervals (rMSSD), and four frequency-domain measures of HVR, including total power (TP; range, <0.40 Hz); low frequency (LF; range, 0.04 to 0.15 Hz), high frequency (HF; range, 0.15 to 0.40 Hz), and LF/HF ratio, were calculated using the continuous data for 24 h recording of the Holter monitor. According to international standards [16,17], rMSSD and HF are assumed to be markers of efferent vagal activity, and SDNN is regarded as a measurement of efferent sympathetic activity, whereas LF is considered to include both sympathetic and vagal modulation, and the LF/HF ratio appears to be a sensitive indicator reflecting sympathovagal balance.

### 2.4. Assessment of Atherosclerosis Conditions in Baroreceptor-Resident Arteries and Coronary Arteries

All patients underwent simultaneous CTAs of the coronary and craniocervical arteries with a 192-slice dual-source computed tomography (Somatom Force; Siemens, Munich, Germany), as previously reported [18]. Two experienced radiologists who were blinded to the clinical information analyzed all the scanned images independently and provided a consensus interpretation of each conclusion.

**Figure 1:**
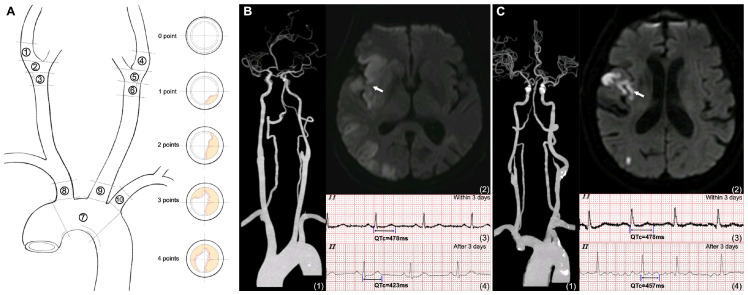
Illustration of TAB-BRA assessment and its indicative value for abnormal repolarization persisting over 3 days after acute ischemic stroke. (**A**) Atherosclerosis conditions in 10 segments of BRA were, respectively, scored with 0 to 4 points according to the percentage of vessel circumference affected by atherosclerosis on orthogonal views (0, none; 1, <25%; 2, 25–49%; 3, 50–74%; 4, ≥75%), then summed as TAB-BRA. (**B**) An acute right insular infarction (white arrow) patient with a low TAB-BRA score (1 point) had a complicated abnormal repolarization (prolonged QTc), and it quickly normalized within 3 days. (**C**) An acute right insular infarction (white arrow) patient with high TAB-BRA scores (17 points) had a complicated abnormal repolarization (prolonged QTc), but it persisted over 3 days, and new atrial fibrillation was detected. (1) Craniocervical computed tomography angiography; (2) brain magnetic resonance imaging of diffusion-weighted image sequence; (3) initial ECG within 3 days of onset; (4) ECG after 3 days of onset. Abbreviations: TAB-BRA = Total Atherosclerosis Burden of Baroreceptor-Resident Arteries.

Atherosclerosis conditions in BRA were quantitatively assessed as TAB-BRA in line with our previous study [10]. Briefly, BRA was divided into 10 segments, including 6 BRA segments in carotid sinuses (bilateral distal common carotid arteries, carotid bifurcations, and origins of internal carotid arteries) and 4 BRA segments in the aortic arch region (aortic arch from the opening of the brachiocephalic trunk to the opening of the left subclavian artery and the origins of three supra-aortic arteries). Atherosclerosis conditions in these 10 segments were, respectively, scored with 0 to 4 points according to the percentage of vessel circumference affected by atherosclerosis on orthogonal views (0, none; 1, <25%; 2, 25–49%; 3, 50–74%; 4, ≥75%), then summed as the TAB-BRA (Figure 1).

The atherosclerosis of coronary arteries was accessed according to the Coronary Artery Disease Reporting and Data System (CAD-RADS) [19]. The 4 main coronary branches—left main, left anterior descending, left circumflex, and right coronary artery, including side branches with a diameter of 1.5 mm or more—were analyzed. The presence of ACAS ≥ 50% was confirmed when there was a stenosis of ≥50% in at least one coronary arterial segment, given that all selected AIS patients had no coronary artery disease history [20].

### 2.5. Statistical Analysis

The baseline characteristics and ECG abnormalities were described and compared by dichotomies of TAB-BRA (≥5 points or <5 points). Continuous variables that followed a normal distribution were presented as mean ± SD and tested by the analysis of a Student’s *t*-test; those that had a skewed distribution were reported as median (interquartile range [IQR]) and compared using Mann–Whitney test. Categorical variables were presented as frequencies (%) and were tested using a chi-square test.

We also performed multivariable logistic regression analyses to investigate associations between TAB-BRA and ECG abnormalities. The adjusted covariates included age, sex, BIM, smoking status, hypertension, diabetes mellitus, prior stroke, initial NIHSS, infarct volume, insular involvement, and ACAS ≥ 50%. A variance inflation factor of <5 was used to test for collinearity. The odds ratio was estimated at 95% CI. In addition, a Spearman correlation analysis and partial correlation analysis would be used to evaluate the correlations between TAB-BRA and HRV parameters. All analyses were implemented in R statistical software version 4.3.1 (R Foundation for Statistical Computing, Vienna, Austria). A 2-tailed *p* value below 0.05 was considered to indicate statistical significance.

## 3. Results

A total of 305 AIS patients were eligible in the initial assessment, but 6 refused to participate; of the 299 included patients, 45 (15.1%) had a prior history of heart diseases. After the further exclusion of patients who met other exclusion criteria (*n* = 26), 228 subjects were eventually analyzed (for the study flow chart, see Supplementary Figure S1).

### 3.1. Baseline Characteristics of the Analyzed AIS Patients

The analyzed patients had an average age of 59 years, and males were predominant (73.7%). The median initial NIHSS score was 3 (2–5) points, and the median infarct volume was 1.9 (0.8–8.7) mL. Insular involvement was observed in 24 (10.5%) patients. There were 101 (44.3%) patients with ACAS ≥ 50%. Concurrently, 198 (86.6%) had atherosclerosis in BRA; the median TAB-BRA score was 5 (1–11) points. Compared with participants with a lower TAB-BRA (<5 points), those with a higher TAB-BRA (≥5 points) were more likely to be older, serious at admission, and have a higher probability of smoking, hypertension, diabetes mellitus, prior stroke, and ACAS ≥ 50% (Table 1).

### 3.2. Associations between TAB-BRA and ECG Abnormalities after AIS

Abnormal repolarization was detected in 113 (49.6%) patients, and SCA in 43 (18.9%). Significantly, abnormal repolarization (62.1% vs. 36.6%, *p* < 0.001) and SCA (30.2% vs. 7.1%, *p* < 0.001) were more frequently observed in patients with a higher TAB-BRA (≥5 points) than a lower TAB-BRA (<5 points). After adjusting for covariates of age, sex, BMI, smoking status, hypertension, diabetes mellitus, prior stroke, initial NIHSS, infarct volume, insular involvement, and ACAS ≥ 50%, logistic regression analyses showed that TAB-BRA was associated with abnormal repolarization (OR 1.09; CI% 1.03–1.16; *p* = 0.003) and SCA (OR 1.08; CI% 1.01–1.15; *p* = 0.021) (Table 2 and Supplementary Table S1).

### 3.3. Indicative Value of TAB-BRA for Abnormal Repolarization Persisting over 3 Days after AIS

Among 113 patients with abnormal repolarization, 65 (57.5%) had abnormal repolarization persisting over 3 days, and logistic regression analyses after adjusting for predetermined variables suggested that TAB-BRA was the strongest independent indicator of abnormal repolarization persisting over 3 days after AIS (OR 1.17; CI% 1.05–1.30; *p* = 0.003) (Table 3). Importantly, we also found that the presence of abnormal repolarization increased the risk of SCA by 4.07 times (CI% 1.58–10.44; *p* = 0.004), but abnormal repolarization persisting over 3 days increased the risk of SCA by 7.24 times (CI% 2.94–17.83; *p* < 0.001) (Supplementary Table S2). The indicative value of TAB-BRA for abnormal repolarization persisting over 3 days after AIS is exemplified in Figure 1.

### 3.4. Relationships of ACAS ≥ 50% and ECG Abnormalities after AIS

**Figure 2:**
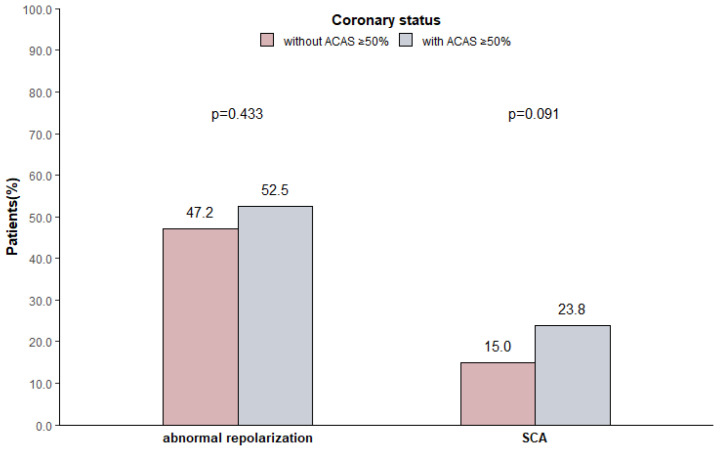
Occurrence of ECG abnormalities in acute ischemic stroke patients with ACAS ≥ 50% and without ACAS ≥ 50%. Abbreviations: ACAS = asymptomatic coronary artery stenosis; SCA = serious cardiac arrhythmias.

There was no apparent difference in the distribution of abnormal repolarization (52.5% vs. 47.2%, *p* = 0.443) and SCA (23.8% vs. 15.0%, *p* = 0.091) between patients with ACAS ≥ 50% and without ACAS ≥ 50% (Figure 2). Considering that the high TAB-BRA group was more likely to have ACAS ≥ 50%, comparisons of ECG abnormalities after AIS between patients with ACAS ≥ 50% and without ACAS ≥ 50% in the high TAB-BRA group and low TAB-BRA group were performed, respectively, but the results still showed no significant difference (Supplementary Figure S2).

### 3.5. Correlations of TAB-BRA and Heart Rate Variability Parameters after AIS

**Figure 3:**
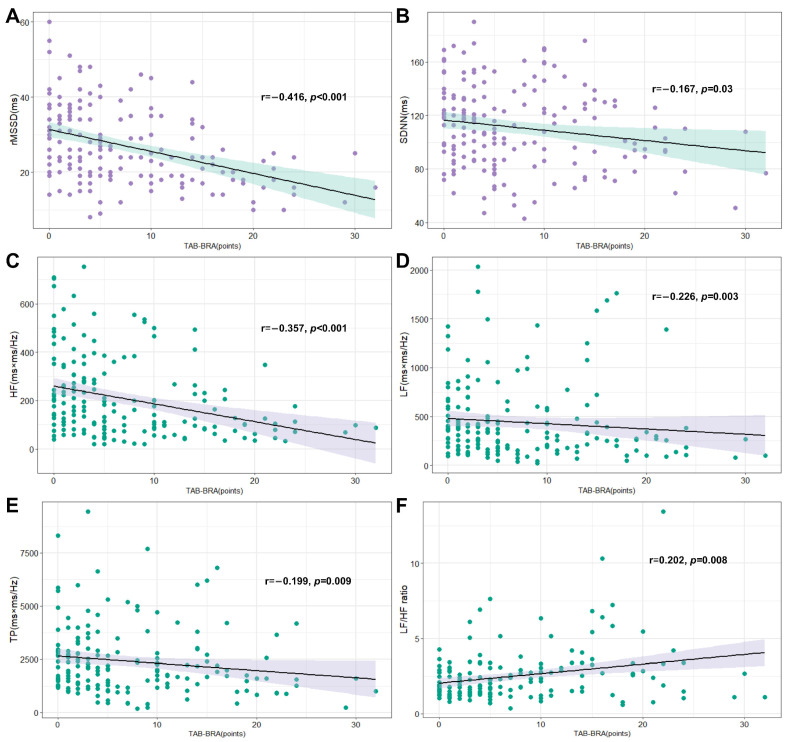
Scatter plots of TAB-BRA and heart rate variability parameters. (**A**,**B**): TAB-BRA vs two time-domain measures (rMSSD and SDNN, respectively), green color indicates 95% confidence interval band. (**C**–**F**): TAB-BRA vs four frequency-domain measures (HF, LF, TP and LF/HF, respectively), purple color indicates 95% confidence interval band. Abbreviations: TAB-BRA = Total Atherosclerosis Burden of Baroreceptor-Resident Arteries; rMSSD = root mean square of successive difference in RR interval; SDNN = SD of all normal-to-normal RR intervals; HF = high frequency; LF = low frequency; and TP = total power.

Spearman correlation analyses illustrated that TAB-BRA was negatively correlated with rMSSD, SDNN, HF, LF, and TP, but positively correlated with the LF/HF ratio (Figure 3). After controlling for age, sex, BMI, smoking status, hypertension, diabetes mellitus, prior stroke, initial NIHSS, infarct volume, insular involvement, and ACAS ≥ 50%, partial correlation analyses showed that TAB-BRA remained significantly correlated with rMSSD (r = −0.334, *p* < 0.001), HF (r = −0.170, *p* = 0.03), and the LF/HF ratio (r = 0.197, *p* = 0.012).

## 4. Discussion

With the atherosclerosis of carotid sinuses, aortic arch, and coronary artery simultaneously evaluated, the present study discovered that a high TAB-BRA was positively associated with abnormal repolarization and SCA in AIS patients without heart disease history and was also the strongest independent indicator of abnormal repolarization persisting over 3 days. However, ACAS ≥ 50% did not relate to abnormal repolarization and SCA. We also found that TAB-BRA was negatively correlated with rMSSD and HF, which are markers of cardiac parasympathetic activity.

To our knowledge, this is the first study to investigate the associations between the atherosclerosis of BRA and ECG abnormalities after AIS. The TAB-BRA was measured in this study to reflect the overall atherosclerosis of BRA. It might provide more information about the influence of atherosclerosis on baroreceptor reflex sensing than merely evaluating the presence or absence of atherosclerosis on just one artery of BRA [21,22,23]. As previously shown by Nasr et al. [21], baroreflex sensitivity impairment was found in bilateral, but not in unilateral, carotid atherosclerosis. A physiological study revealed that the atherosclerosis of the aortic arch, which displays a higher mechanosensitivity than carotid sinuses, could also diminish the sensitivity of baroreceptors [22,23]. This research hinted that a method to comprehensively characterize the atherosclerosis of BRA might build a more reliable connection between atherosclerosis degree and baroreflex impairments. Therefore, the measurement of TAB-BRA offered potential advantages in revealing the relationship between the atherosclerosis of BRA and ECG abnormalities after AIS.

Our results demonstrated that AIS patients with a higher TAB-BRA were more predisposed to abnormal repolarization and SCA. Abnormal repolarization is the most common type of ECG abnormality after AIS and has been linked to the occurrence of cardiac arrhythmias and myocardial injury in AIS patients [1]. SCA may impact the prognosis of AIS patients by hemodynamic instability and sudden cardiac death [15]. Established influencing factors of these ECG abnormalities after AIS also include cardiovascular risk factors, premorbid cardiac disease, and stroke features, such as stroke severity, infarct volumes, and insular involvement [1,24,25]. Our study showed that AIS patients with a higher TAB-BRA were more likely to be older in age, smoke, and have hypertension, diabetes mellitus, and a prior stroke. Whether or not these confounding factors were involved in the association between TAB-BRA and ECG abnormalities deserves consideration. However, after adjustment for these cofactors, stroke features, and ACAS ≥ 50%, TAB-BRA was still positively associated with abnormal repolarization and SCA in AIS patients without a pre-existing heart disease. The possible explanation for these results may be that those AIS patients with a higher TAB-BRA might have less physiological baroreflex reserve, and poor baroreflex regulation of cardiovascular activity makes them more vulnerable to abnormal repolarization and SCA.

We further found that a high TAB-BRA might independently predict abnormal repolarization persisting over 3 days. Previous data on the time course of ECG abnormalities during the acute phase of ischemic stroke displayed that abnormal repolarization occurred predominantly within the first 3 days after AIS [2]. There is evidence that persistent ECG abnormalities are more strongly associated with an unfavorable outcome [1,26]. For instance, Milan et al. [26] found that those AIS patients whose QTc was prolonged to the third day after stroke had a much higher risk of poor neurological function and mortality. Thus, it is of clinical significance to determine the factors influencing the duration of ECG abnormalities, but it has not yet been studied before. In our cohort, 49.6% of AIS patients had an abnormal repolarization upon hospital admission, and this proportion remained at 28.5% after 3 days of onset. Furthermore, while AIS patients with an abnormal repolarization had a 4.07 times higher risk of SCA, abnormal repolarization persisting over 3 days increased the risk of SCA by 7.24 times. As exemplified in Figure 3, abnormal repolarization was detected in two AIS patients with insular involvement, when normalized within 3 days after onset in the patient with a low TAB-BRA (1 point), but persisting over 3 days after onset in the patient with a high TAB-BRA (17 points), and new atrial fibrillation was also detected. This result suggests that evaluating the atherosclerosis of BRA may provide additional evidence for intensive ECG monitoring for at least 72 h and an early prevention of high-risk ECG abnormalities after AIS.

While known coronary artery disease is usually considered a significant cause of ECG abnormalities in AIS patients, the impact of subclinical coronary artery disease (ACAS ≥ 50%) is still unknown, which is critical for our study to understand the relationship between TAB-BRA and ECG abnormalities. Previous studies reported that approximately 18% to 48% of patients with AIS had ACAS ≥ 50%, and those with carotid atherosclerotic stenosis were more likely to have ACAS ≥ 50% [20,27,28]. In the present study, 44.3% of patients had ACAS ≥50%, and patients with a high TAB-BRA had more ACAS ≥ 50% than those with a low TAB-BRA (62.9% vs. 25%, *p* < 0.001). This result is also consistent with a previous study, which found that TAB-BRA could identify patients with heavy coronary atherosclerosis [29]. Of note, our results found no correlation between ACAS ≥ 50% and abnormal repolarization and SCA. This indicates that these ECG abnormalities during the acute stage of ischemic stroke may be less likely caused by subclinical coronary artery disease, and suggests the significance of exploring the neurogenically mediated nonischemic mechanisms of TAB-BRA on cardiac electrical instability after AIS.

HRV was commonly used to evaluate cardiac autonomic nervous system dysfunction after AIS, which has been proven to play a major role in ECG abnormalities after AIS [16,17]. In our study, we found that TAB-BRA was negatively related to the HRV parameters reflecting the vagal component of cardiovascular regulation. A previous study that also observed a relative decrease in the parasympathetic component of HRV in bilateral carotid atherosclerosis provided some support for our findings [21]. Of note, in most cases, sympathetic hyperactivity can increase the occurrence of arrhythmia, while the parasympathetic nervous system can inhibit its occurrence [30]. Physiologically, when baroreceptors of BRA sense the stretch of the arterial wall, increased afferent impulses are relayed to the nucleus tractus solitarius of the brainstem, which then induces the activation of parasympathetic activity and the relative inhibition of sympathetic tone [31]. The atherosclerosis of BRA in AIS patients may blunt the stretch of the baroreceptors, leading to a diminished neural input to brainstem autonomic control centers and thereby to decreased parasympathetic output to the cardiovascular system [9,10]. In addition, our data also showed a positive relationship between TAB-BRA and the LH/HF ratio, an accurate marker of the shifts in sympathovagal balance [16]. Therefore, it can be inferred from our results that AIS patients with a higher TAB-BRA tend to have a lower cardiac parasympathetic activity, which further aggravates the stroke-induced autonomic imbalance, then puts them at a higher risk of abnormal repolarization and SCA. This implies that strengthening the evaluation and prevention of the atherosclerosis of BRA in AIS patients may be significant in improving cardiac autonomic and heart rate regulation and reducing the risk of ECG abnormalities after AIS. Future studies are warranted to validate its value.

## 5. Limitations

There are also some limitations. First, as a single-center study with a limited sample size, caution must be taken in the generalizability of our results. Second, it remains a challenge to distinguish whether the ECG abnormalities detected after AIS are new events induced by AIS or pre-existing conditions before stroke onset. Compared to previous studies, our study was conducted in AIS patients without a prior heart disease, we and further considered whether there was ACAS ≥50%, addressing this problem as much as possible. Third, the neurological deficits (the median NIHSS at admission was 3 points) of the patients were relatively minor, so the incidence of ECG abnormalities in our study may be underestimated. Lastly, the long-term outcomes of abnormal repolarization and SCA after AIS are unclear; further research is needed to clarify this issue.

## 6. Conclusions

AIS patients with a high TAB-BRA are more likely to have high risks of abnormal repolarization and SCA and abnormal repolarization persisting over 3 days. These risks may be related to the decreased cardiac parasympathetic activity caused by a high TAB-BRA, but not related to the accompanying ACAS ≥50%. Our findings may not only open a novel perspective for exploring new mechanisms of ECG abnormalities after AIS, but also lay the foundations for developing pathophysiology-based new targets for the prevention and treatment of these ECG abnormalities.

## Figures and Tables

**Table 1:** Baseline characteristics of the analyzed acute ischemic stroke patients.

Characteristics	Total (*n* = 228)	TAB-BRA < 5 (*n* = 112)	TAB-BRA ≥ 5 (*n* = 116)	*p* Value
Demographics				
Age, y, mean ± SD	59.53 ± 12.10	53.8 ± 12.1	65.0 ± 9.3	<0.001
Male, *n* (%)	168 (73.7)	79 (70.5)	89 (76.7)	0.289
BMI, kg/m^2^, mean ± SD	25.79 ± 3.54	26.1 ± 3.9	25.4 ± 3.2	0.139
Smoking, *n* (%)	115(50.4)	49 (43.8)	66 (56.9)	0.047
Medical histories				
Hypertension, *n* (%)	140 (61.4)	56 (50.0)	84 (72.4)	<0.001
Diabetes mellitus, *n* (%)	79 (34.6)	25 (22.3)	54 (46.6)	<0.001
Prior stroke, *n* (%)	47 (20.6)	10 (8.9)	37 (31.9)	<0.001
Stroke features				
Initial NIHSS, median (IQR)	3 (2–5)	3 (2–4)	4 (2,5)	<0.001
Infarct volume, mL, median (IQR)	1.9 (0.8–8.7)	2.1 (0.8–8.6)	1.7 (0.8–10.3)	0.926
Insular involvement, *n* (%)	24 (10.5)	12 (10.7)	12 (10.3)	0.928
Coronary status				
ACAS ≥ 50%, *n* (%)	101 (44.3)	28 (25.0)	73 (62.9)	<0.001
Treatment, *n* (%)				
Antiplatelet	223 (97.8)	109 (97.3)	114 (98.3)	0.832
Anticoagulation	14 (6.1)	6 (5.4)	8 (6.9)	0.796
Thrombolysis	8 (3.5)	4 (3.6)	4 (3.4)	0.921

Abbreviations: TAB-BRA = Total Atherosclerosis Burden of Baroreceptor-Resident Arteries; SD = standard deviation; BMI = body mass index; NIHSS = National Institutes of Health Stroke Scale; IQR = interquartile range; and ACAS = asymptomatic coronary artery stenosis.

**Table 2:** Adjusted odds ratio of TAB-BRA for ECG abnormalities after acute ischemic stroke.

Indices	Adjusted OR (95% CI) †	*p* Value
Abnormal repolarization	1.09 (1.03–1.16)	0.003
SCA	1.08 (1.01–1.15)	0.021

Abbreviations: TAB-BRA = Total Atherosclerosis Burden of Baroreceptor-Resident Arteries; OR = odds ratio; CI = confidence interval; SCA = serious cardiac arrhythmias. † OR of TAB-BRA were adjusted for the following covariates: age, sex, body mass index, smoking status, hypertension, diabetes mellitus, prior stroke, initial NIHSS, infarct volume, insular involvement, and asymptomatic coronary artery stenosis ≥50%.

**Table 3:** Univariate and multivariate logistic regression analysis for abnormal repolarization persisting over 3 days after acute ischemic stroke.

Variable	Abnormal Repolarization Persisting over 3 Days			
	Crude OR (95% CI)	*p* Value	Adjusted OR (95% CI) †	*p* Value
Age, year	1.10 (1.05, 1.16) *	<0.001	1.06 (1.00, 1.13)	0.069
Sex (male)	1.02 (0.46, 2.29)	0.956	1.40 (0.35, 5.57)	0.633
BMI, kg/m^2^	0.96 (0.86, 1.06)	0.427	0.90 (0.77, 1.05)	0.167
Smoking status	0.84 (0.40, 1.77)	0.645	0.35 (0.10, 1.27)	0.110
Hypertension	2.39 (1.08, 5.28) *	0.031	0.81 (0.27, 2.47)	0.711
Diabetes mellitus	1.56 (0.73, 3.36)	0.254	0.66 (0.23, 1.88)	0.438
Prior stroke	2.89 (1.05, 7.93) *	0.039	1.18 (0.32, 4.39)	0.804
Initial NIHSS	1.09 (0.94, 1.27)	0.267	1.17 (0.93, 1.46)	0.182
Infarct volume, mL	0.99 (0.97, 1.00)	0.149	0.99 (0.96, 1.01)	0.208
Insular involvement	0.34 (0.13, 0.90) *	0.029	0.31 (0.07, 1.37)	0.122
ACAS ≥ 50%	3.64 (1.64, 8.07) *	0.001	2.71 (0.85, 8.64)	0.091
TAB-BRA, points	1.22 (1.12, 1.33) *	<0.001	1.17 (1.05, 1.30) *	0.003

Abbreviations: TAB-BRA = Total Atherosclerosis Burden of Baroreceptor-Resident Arteries; OR = odds ratio; CI = confidence interval; BMI = body mass index; NIHSS = National Institutes of Health Stroke Scale; and ACAS = asymptomatic coronary artery stenosis. † ORs were adjusted for the following covariates: age, sex, body mass index, smoking status, hypertension, diabetes mellitus, prior stroke, initial NIHSS, infarct volume, insular involvement, and asymptomatic coronary artery stenosis ≥50%. * indicates *p* < 0.05.

## Data Availability

Due to privacy and ethical reasons, the data supporting the findings of this study are available from the corresponding author for checking the reproducibility of the study results.

## Supplementary Materials

The following supporting information can be downloaded at https://www.mdpi.com/article/10.3390/brainsci14050505/s1, Figure S1. Study flow chart. Figure S2: Comparisons of ECG abnormalities after acute ischemic stroke between patients with ACAS ≥50% and without ACAS ≥50% in the high TAB-BRA group and low TAB-BRA group. Table S1. Univariate logistic regression analysis for ECG abnormalities after acute ischemic stroke. Table S2. Multivariable logistic analysis of associations between abnormal repolarization and serious cardiac arrhythmias.
