# Supplementary material for: Associations between Total Atherosclerosis Burden of Baroreceptor-Resident Arteries and ECG Abnormalities after Acute Ischemic Stroke
Source: Brain Sci. 2024 May 16;14(5):505. doi: 10.3390/brainsci14050505 (PMC11118932; doi:10.3390/brainsci14050505)
Supplement: Supplementary file 1 [file brainsci-14-00505-s001.zip › Supplemental Files/Supplemental Figures 1-2.pdf]

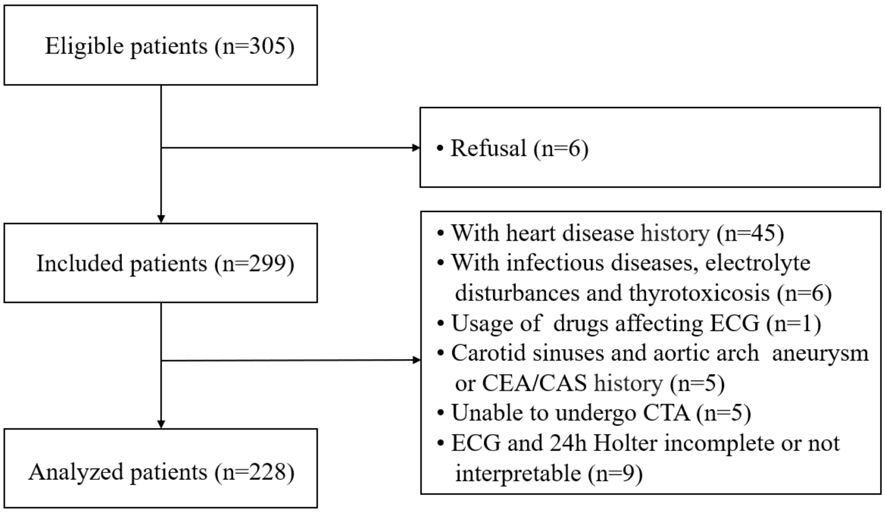

Fig. S1 Study flow chart.

Abbreviations: ECG = electrocardiogram; CEA = carotid endarterectomy; CAS = carotid artery stenting; CTA = computed tomography angiogram.

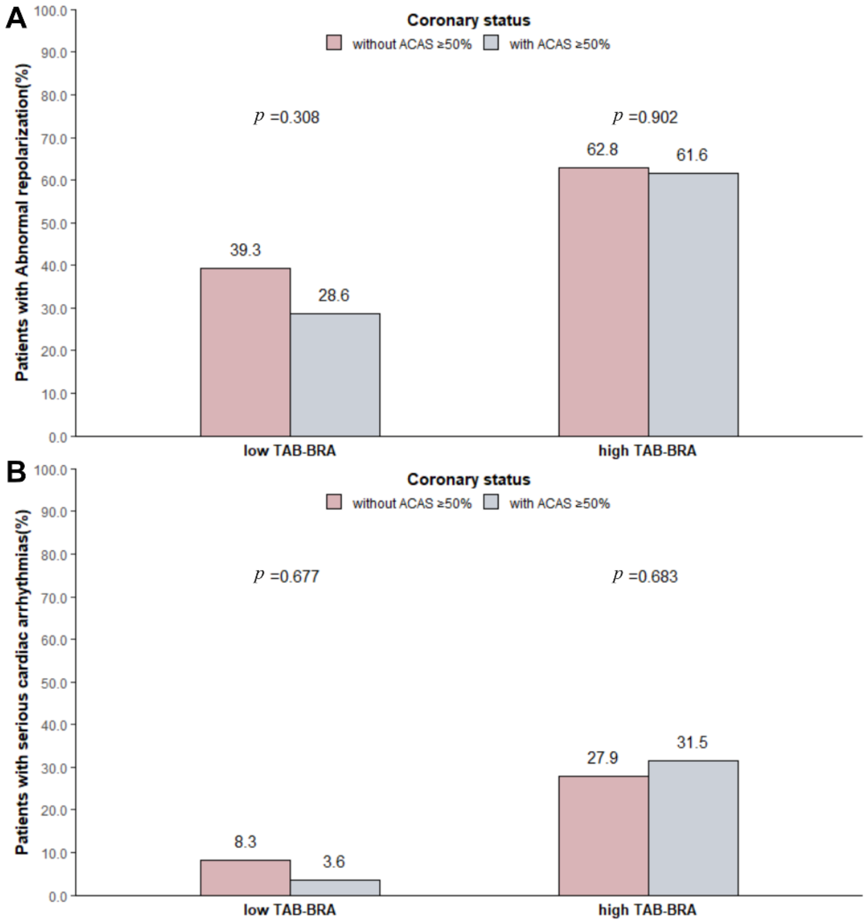

Fig. S2 Comparisons of ECG abnormalities after acute ischemic stroke between patients with ACAS ≥50% and without ACAS ≥50% in the high TAB-BRA group and low TAB-BRA group.

Abbreviations: ACAS = asymptomatic coronary artery stenosis; SCA = serious cardiac arrhythmias.
