# Supplementary material for: Associations between Total Atherosclerosis Burden of Baroreceptor-Resident Arteries and ECG Abnormalities after Acute Ischemic Stroke
Source: Brain Sci. 2024 May 16;14(5):505. doi: 10.3390/brainsci14050505 (PMC11118932; doi:10.3390/brainsci14050505)
Supplement: Supplementary file 1 [file brainsci-14-00505-s001.zip › Supplemental Files/Supplemental Tables 1-2.pdf]

Supplemental Table 1. Univariate logistic regression analysis for ECG abnormalities after acute ischemic stroke

Variable	Crude OR (95% CI)	
	Abnormal repolarization	SCA
Age, year	**1.03 (1.01, 1.06)** *	**1.09 (1.05, 1.13)** *
Sex (male)	0.62 (0.34, 1.12)	1.22 (0.56, 2.66)
BMI, kg/m2	1.02 (0.94, 1.09)	0.93 (0.84, 1.02)
Smoking	0.93 (0.55, 1.57)	1.30 (0.67, 2.54)
Hypertension	1.41 (0.82, 2.41)	1.80 (0.87, 3.73)
Diabetes mellitus	**1.85 (1.06, 3.21)** *	1.15 (0.58, 2.29)
Prior stroke	1.20 (0.63, 2.28)	1.66 (0.77, 3.55)
Initial NIHSS	**1.42 (1.23, 1.65)** *	**1.23 (1.07, 1.41)** *
Infarct Volume, mL	1.00 (0.99, 1.01)	1.00 (1.00, 1.01)
Insular involvement	**13.66 (3.13, 59.59)** *	**3.70 (1.52, 9.04)** *
ACAS⩾50%	1.23 (0.73, 2.08)	1.77 (0.91, 3.46)
TAB-BRA, points	**1.09 (1.05, 1.14)** *	**1.12 (1.07, 1.18)** *

Abbreviations: TAB-BRA = Total Atherosclerosis Burden of Baroreceptor-Resident Arteries; OR = odds ratio; CI = confidence interval; BMI = body mass index; NIHSS = National Institutes of Health Stroke Scale; ACAS = asymptomatic coronary artery stenosis; SCA = serious cardiac arrhythmias.

* indicate $p<0.05$.

Supplemental Table 2. Multivariable logistic analysis of associations between abnormal repolarization and serious cardiac arrhythmias

Indices	Adjusted OR (95% CI) †	*p* value
Abnormal repolarization	4.07 (1.58-10.44)	0.004
Abnormal repolarization persisting over 3 days	7.24 (2.94-17.83)	<0.001

Abbreviations: OR = odds ratio; CI = confidence interval.

†ORs were adjusted for the following covariates: age, sex, body mass index, smoking, hypertension, diabetes mellitus, prior stroke, initial NIHSS, infarct Volume, insular involvement, asymptomatic coronary artery stenosis ≥50%.
